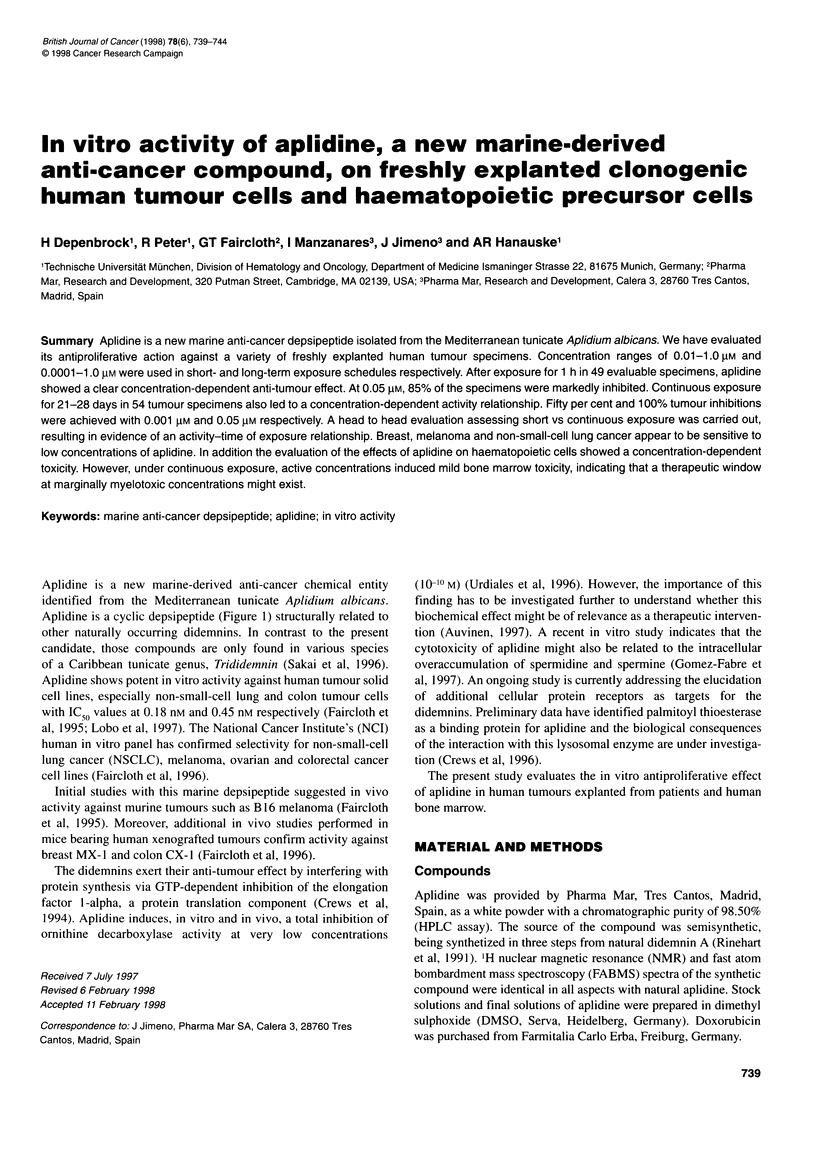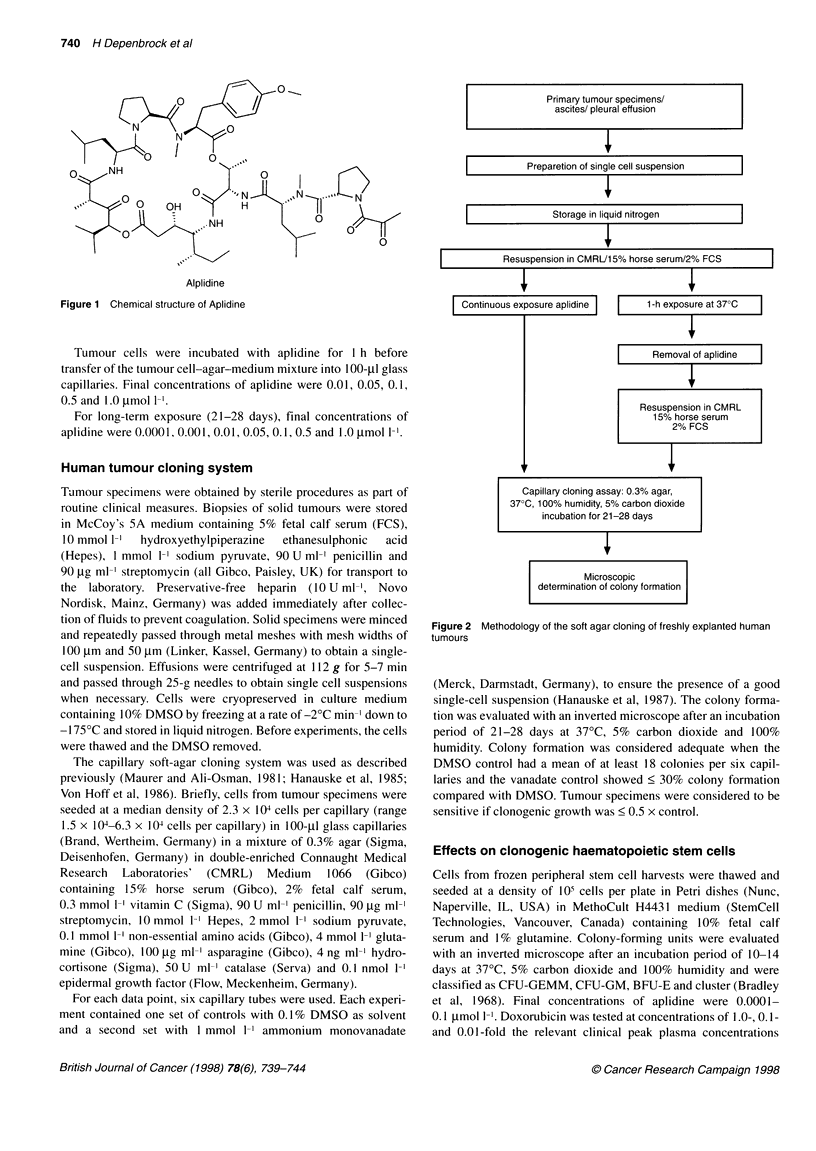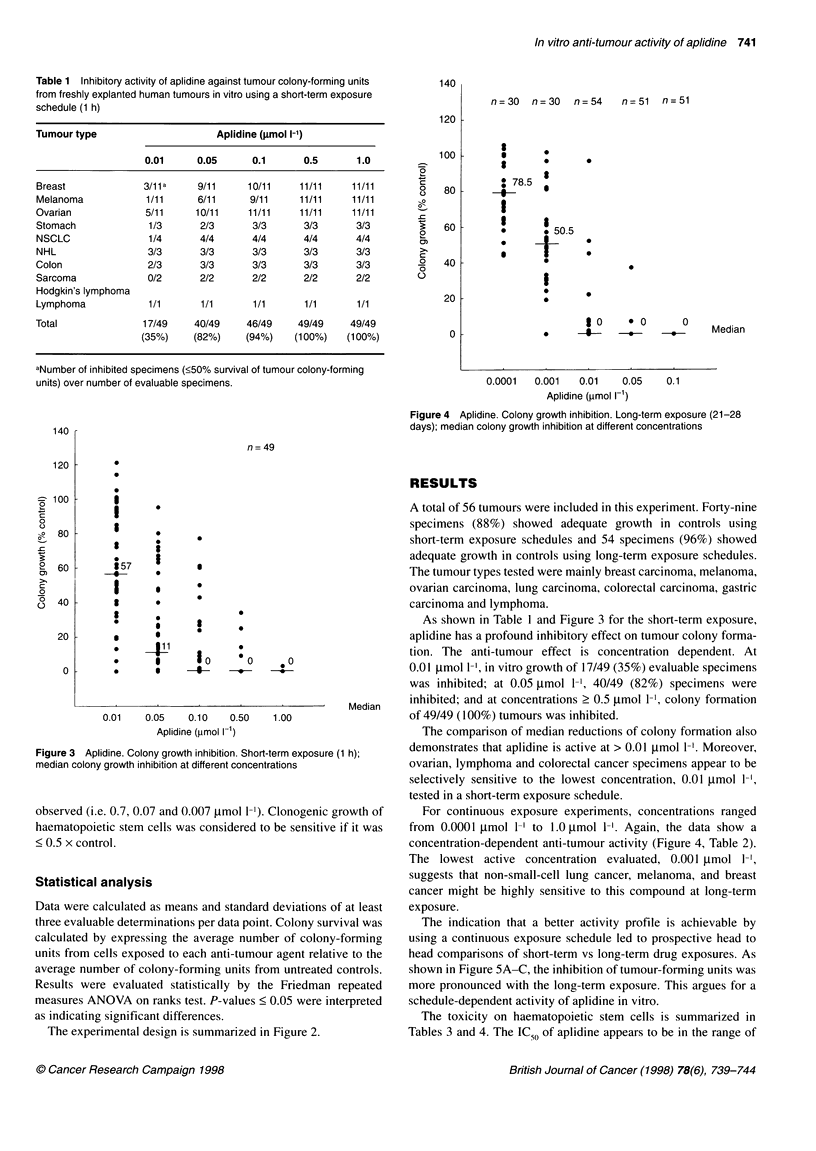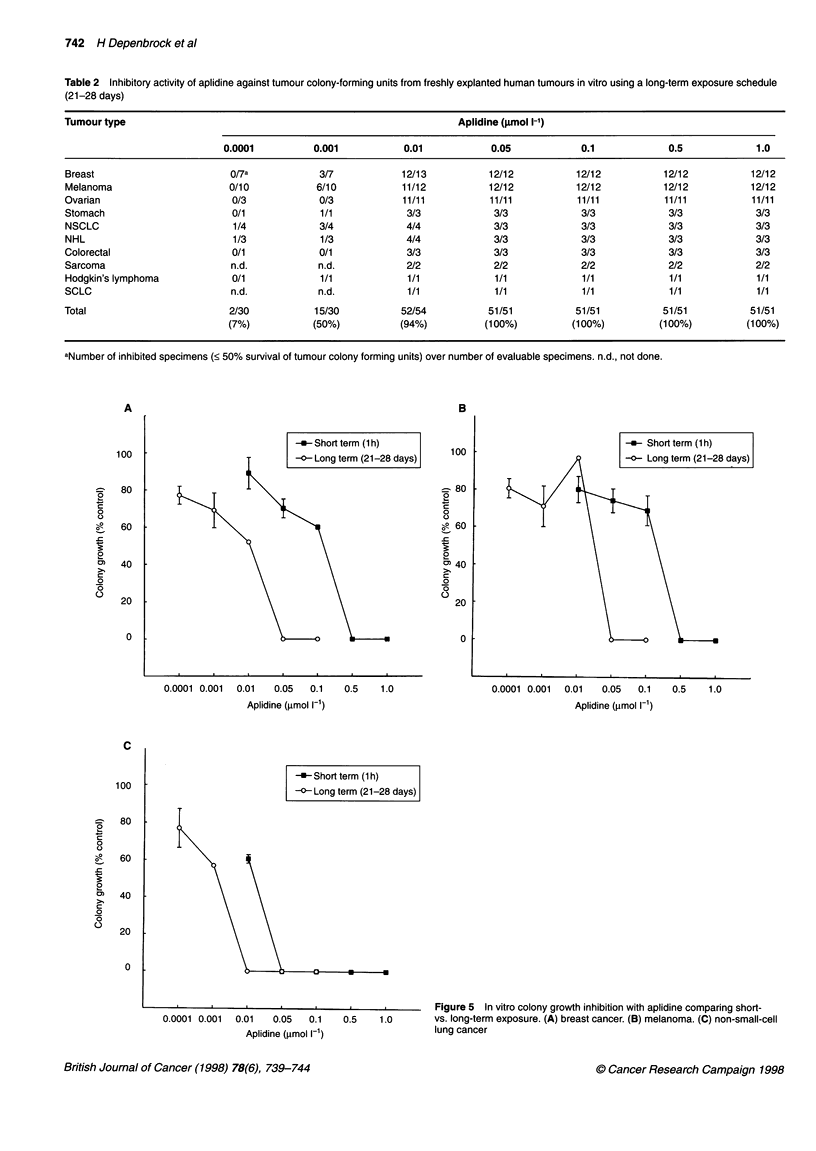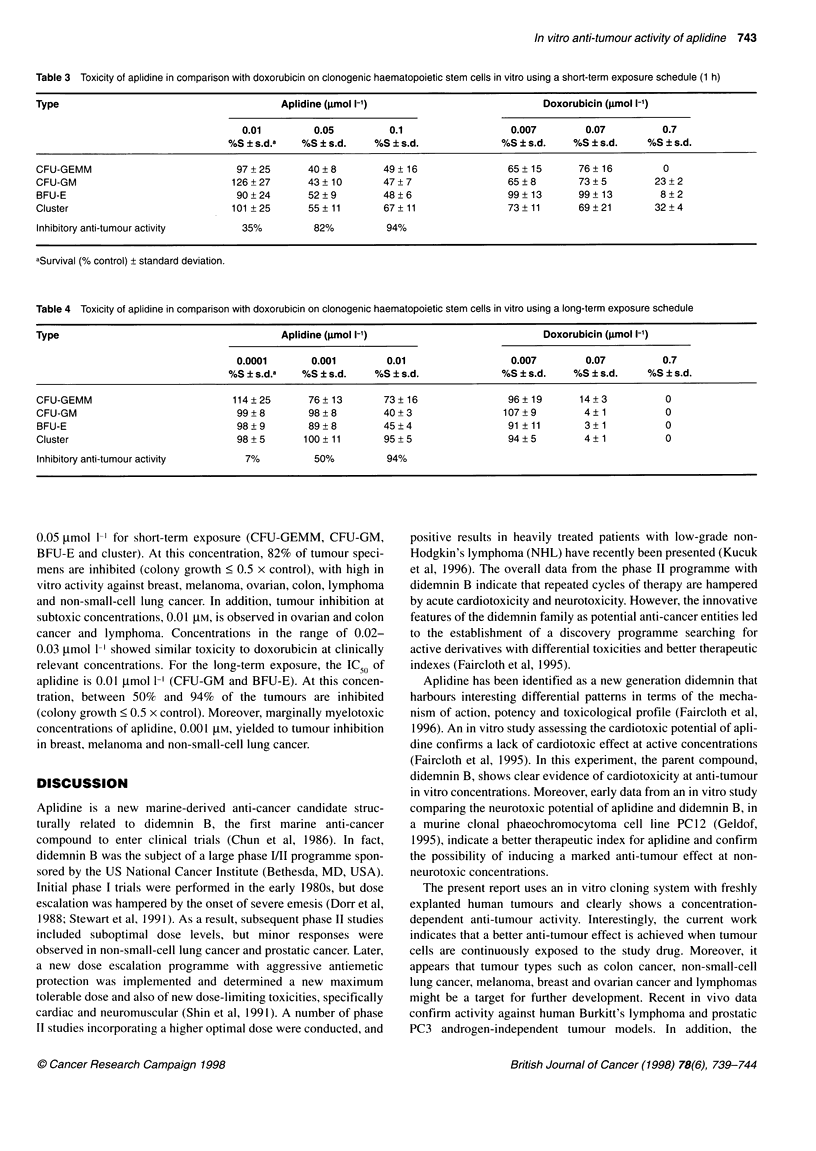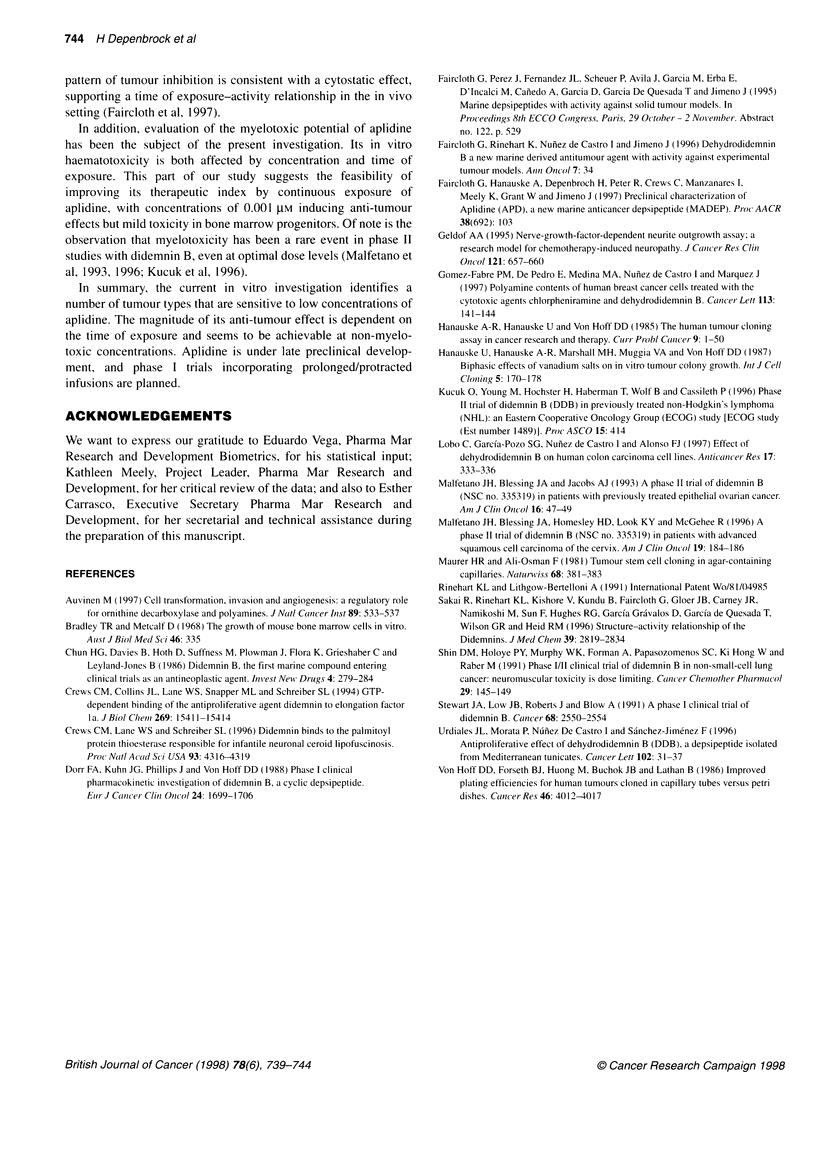# In vitro activity of aplidine, a new marine-derived anti-cancer compound, on freshly explanted clonogenic human tumour cells and haematopoietic precursor cells.

**DOI:** 10.1038/bjc.1998.570

**Published:** 1998-09

**Authors:** H. Depenbrock, R. Peter, G. T. Faircloth, I. Manzanares, J. Jimeno, A. R. Hanauske

**Affiliations:** Technische Universität München, Department of Medicine, Munich, Germany.

## Abstract

Aplidine is a new marine anti-cancer depsipeptide isolated from the Mediterranean tunicate Aplidium albicans. We have evaluated its antiproliferative action against a variety of freshly explanted human tumour specimens. Concentration ranges of 0.01-1.0 microM and 0.0001-1.0 microM were used in short- and long-term exposure schedules respectively. After exposure for 1 h in 49 evaluable specimens, aplidine showed a clear concentration-dependent anti-tumour effect. At 0.05 microM, 85% of the specimens were markedly inhibited. Continuous exposure for 21-28 days in 54 tumour specimens also led to a concentration-dependent activity relationship. Fifty per cent and 100% tumour inhibitions were achieved with 0.001 microM and 0.05 microM respectively. A head to head evaluation assessing short vs continuous exposure was carried out, resulting in evidence of an activity-time of exposure relationship. Breast, melanoma and non-small-cell lung cancer appear to be sensitive to low concentrations of aplidine. In addition the evaluation of the effects of aplidine on haematopoietic cells showed a concentration-dependent toxicity. However, under continuous exposure, active concentrations induced mild bone marrow toxicity, indicating that a therapeutic window at marginally myelotoxic concentrations might exist.


British Journal of Cancer (1998) 78(6), 739–744
© 1998 Cancer Research Campaign

### In vitro activity of aplidine, a new marine-derived anti-cancer compound, on freshly explanted clonogenic human tumour cells and haematopoietic precursor cells

H Depenbrock[1], R Peter[1], GT Faircloth[2], I Manzanares[3], J Jimeno[3] and AR Hanauske[1]

[1]Technische Universität München, Division of Hematology and Oncology, Department of Medicine Ismaninger Strasse 22, 81675 Munich, Germany; [2]Pharma Mar, Research and Development, 320 Putman Street, Cambridge, MA 02139, USA; [3]Pharma Mar, Research and Development, Calera 3, 28760 Tres Cantos, Madrid, Spain

**Summary** Aplidine is a new marine anti-cancer depsipeptide isolated from the Mediterranean tunicate *Aplidium albicans*. We have evaluated its antiproliferative action against a variety of freshly explanted human tumour specimens. Concentration ranges of 0.01–1.0 μM and 0.0001–1.0 μM were used in short- and long-term exposure schedules respectively. After exposure for 1 h in 49 evaluable specimens, aplidine showed a clear concentration-dependent anti-tumour effect. At 0.05 μM, 85% of the specimens were markedly inhibited. Continuous exposure for 21–28 days in 54 tumour specimens also led to a concentration-dependent activity relationship. Fifty per cent and 100% tumour inhibitions were achieved with 0.001 μM and 0.05 μM respectively. A head to head evaluation assessing short vs continuous exposure was carried out, resulting in evidence of an activity–time of exposure relationship. Breast, melanoma and non-small-cell lung cancer appear to be sensitive to low concentrations of aplidine. In addition the evaluation of the effects of aplidine on haematopoietic cells showed a concentration-dependent toxicity. However, under continuous exposure, active concentrations induced mild bone marrow toxicity, indicating that a therapeutic window at marginally myelotoxic concentrations might exist.

**Keywords:** marine anti-cancer depsipeptide; aplidine; in vitro activity

Aplidine is a new marine-derived anti-cancer chemical entity identified from the Mediterranean tunicate *Aplidium albicans*. Aplidine is a cyclic depsipeptide (Figure 1) structurally related to other naturally occurring didemnins. In contrast to the present candidate, those compounds are only found in various species of a Caribbean tunicate genus, *Trididemnin* (Sakai et al, 1996). Aplidine shows potent in vitro activity against human tumour solid cell lines, especially non-small-cell lung and colon tumour cells with $IC_{50}$ values at 0.18 nM and 0.45 nM respectively (Faircloth et al, 1995; Lobo et al, 1997). The National Cancer Institute's (NCI) human in vitro panel has confirmed selectivity for non-small-cell lung cancer (NSCLC), melanoma, ovarian and colorectal cancer cell lines (Faircloth et al, 1996).

Initial studies with this marine depsipeptide suggested in vivo activity against murine tumours such as B16 melanoma (Faircloth et al, 1995). Moreover, additional in vivo studies performed in mice bearing human xenografted tumours confirm activity against breast MX-1 and colon CX-1 (Faircloth et al, 1996).

The didemnins exert their anti-tumour effect by interfering with protein synthesis via GTP-dependent inhibition of the elongation factor 1-alpha, a protein translation component (Crews et al, 1994). Aplidine induces, in vitro and in vivo, a total inhibition of ornithine decarboxylase activity at very low concentrations ($10^{-10}$ M) (Urdiales et al, 1996). However, the importance of this finding has to be investigated further to understand whether this biochemical effect might be of relevance as a therapeutic intervention (Auvinen, 1997). A recent in vitro study indicates that the cytotoxicity of aplidine might also be related to the intracellular overaccumulation of spermidine and spermine (Gomez-Fabre et al, 1997). An ongoing study is currently addressing the elucidation of additional cellular protein receptors as targets for the didemnins. Preliminary data have identified palmitoyl thioesterase as a binding protein for aplidine and the biological consequences of the interaction with this lysosomal enzyme are under investigation (Crews et al, 1996).

The present study evaluates the in vitro antiproliferative effect of aplidine in human tumours explanted from patients and human bone marrow.

*Received 7 July 1997*
*Revised 6 February 1998*
*Accepted 11 February 1998*

*Correspondence to:* J Jimeno, Pharma Mar SA, Calera 3, 28760 Tres Cantos, Madrid, Spain

### MATERIAL AND METHODS

### Compounds

Aplidine was provided by Pharma Mar, Tres Cantos, Madrid, Spain, as a white powder with a chromatographic purity of 98.50% (HPLC assay). The source of the compound was semisynthetic, being synthetized in three steps from natural didemnin A (Rinehart et al, 1991). $^1$H nuclear magnetic resonance (NMR) and fast atom bombardment mass spectroscopy (FABMS) spectra of the synthetic compound were identical in all aspects with natural aplidine. Stock solutions and final solutions of aplidine were prepared in dimethyl sulphoxide (DMSO, Serva, Heidelberg, Germany). Doxorubicin was purchased from Farmitalia Carlo Erba, Freiburg, Germany.

Aplidine

**Figure 1** Chemical structure of Aplidine

**Figure 2** Methodology of the soft agar cloning of freshly explanted human tumours

Tumour cells were incubated with aplidine for 1 h before transfer of the tumour cell–agar–medium mixture into 100-μl glass capillaries. Final concentrations of aplidine were 0.01, 0.05, 0.1, 0.5 and 1.0 μmol $l^{-1}$.

For long-term exposure (21–28 days), final concentrations of aplidine were 0.0001, 0.001, 0.01, 0.05, 0.1, 0.5 and 1.0 μmol $l^{-1}$.

### Human tumour cloning system

Tumour specimens were obtained by sterile procedures as part of routine clinical measures. Biopsies of solid tumours were stored in McCoy's 5A medium containing 5% fetal calf serum (FCS), 10 mmol $l^{-1}$ hydroxyethylpiperazine ethanesulphonic acid (Hepes), 1 mmol $l^{-1}$ sodium pyruvate, 90 U $ml^{-1}$ penicillin and 90 μg $ml^{-1}$ streptomycin (all Gibco, Paisley, UK) for transport to the laboratory. Preservative-free heparin (10 U $ml^{-1}$, Novo Nordisk, Mainz, Germany) was added immediately after collection of fluids to prevent coagulation. Solid specimens were minced and repeatedly passed through metal meshes with mesh widths of 100 μm and 50 μm (Linker, Kassel, Germany) to obtain a single-cell suspension. Effusions were centrifuged at 112 *g* for 5–7 min and passed through 25-g needles to obtain single cell suspensions when necessary. Cells were cryopreserved in culture medium containing 10% DMSO by freezing at a rate of −2°C $min^{-1}$ down to −175°C and stored in liquid nitrogen. Before experiments, the cells were thawed and the DMSO removed.

The capillary soft-agar cloning system was used as described previously (Maurer and Ali-Osman, 1981; Hanauske et al, 1985; Von Hoff et al, 1986). Briefly, cells from tumour specimens were seeded at a median density of $2.3 \times 10^4$ cells per capillary (range $1.5 \times 10^4$–$6.3 \times 10^4$ cells per capillary) in 100-μl glass capillaries (Brand, Wertheim, Germany) in a mixture of 0.3% agar (Sigma, Deisenhofen, Germany) in double-enriched Connaught Medical Research Laboratories' (CMRL) Medium 1066 (Gibco) containing 15% horse serum (Gibco), 2% fetal calf serum, 0.3 mmol $l^{-1}$ vitamin C (Sigma), 90 U $ml^{-1}$ penicillin, 90 μg $ml^{-1}$ streptomycin, 10 mmol $l^{-1}$ Hepes, 2 mmol $l^{-1}$ sodium pyruvate, 0.1 mmol $l^{-1}$ non-essential amino acids (Gibco), 4 mmol $l^{-1}$ glutamine (Gibco), 100 μg $ml^{-1}$ asparagine (Gibco), 4 ng $ml^{-1}$ hydrocortisone (Sigma), 50 U $ml^{-1}$ catalase (Serva) and 0.1 nmol $l^{-1}$ epidermal growth factor (Flow, Meckenheim, Germany).

For each data point, six capillary tubes were used. Each experiment contained one set of controls with 0.1% DMSO as solvent and a second set with 1 mmol $l^{-1}$ ammonium monovanadate (Merck, Darmstadt, Germany), to ensure the presence of a good single-cell suspension (Hanauske et al, 1987). The colony formation was evaluated with an inverted microscope after an incubation period of 21–28 days at 37°C, 5% carbon dioxide and 100% humidity. Colony formation was considered adequate when the DMSO control had a mean of at least 18 colonies per six capillaries and the vanadate control showed ≤ 30% colony formation compared with DMSO. Tumour specimens were considered to be sensitive if clonogenic growth was ≤ 0.5 × control.

### Effects on clonogenic haematopoietic stem cells

Cells from frozen peripheral stem cell harvests were thawed and seeded at a density of $10^5$ cells per plate in Petri dishes (Nunc, Naperville, IL, USA) in MethoCult H4431 medium (StemCell Technologies, Vancouver, Canada) containing 10% fetal calf serum and 1% glutamine. Colony-forming units were evaluated with an inverted microscope after an incubation period of 10–14 days at 37°C, 5% carbon dioxide and 100% humidity and were classified as CFU-GEMM, CFU-GM, BFU-E and cluster (Bradley et al, 1968). Final concentrations of aplidine were 0.0001–0.1 μmol $l^{-1}$. Doxorubicin was tested at concentrations of 1.0-, 0.1- and 0.01-fold the relevant clinical peak plasma concentrations

© Cancer Research Campaign 1998

**Table 1** Inhibitory activity of aplidine against tumour colony-forming units from freshly explanted human tumours in vitro using a short-term exposure schedule (1 h)

| **Tumour type** | **Aplidine (µmol l⁻¹)** | | | | |
|---|---|---|---|---|---|
| | **0.01** | **0.05** | **0.1** | **0.5** | **1.0** |
| Breast | 3/11[a] | 9/11 | 10/11 | 11/11 | 11/11 |
| Melanoma | 1/11 | 6/11 | 9/11 | 11/11 | 11/11 |
| Ovarian | 5/11 | 10/11 | 11/11 | 11/11 | 11/11 |
| Stomach | 1/3 | 2/3 | 3/3 | 3/3 | 3/3 |
| NSCLC | 1/4 | 4/4 | 4/4 | 4/4 | 4/4 |
| NHL | 3/3 | 3/3 | 3/3 | 3/3 | 3/3 |
| Colon | 2/3 | 3/3 | 3/3 | 3/3 | 3/3 |
| Sarcoma | 0/2 | 2/2 | 2/2 | 2/2 | 2/2 |
| Hodgkin's lymphoma | | | | | |
| Lymphoma | 1/1 | 1/1 | 1/1 | 1/1 | 1/1 |
| Total | 17/49 (35%) | 40/49 (82%) | 46/49 (94%) | 49/49 (100%) | 49/49 (100%) |

[a]Number of inhibited specimens (≤50% survival of tumour colony-forming units) over number of evaluable specimens.

**Figure 3** Aplidine. Colony growth inhibition. Short-term exposure (1 h); median colony growth inhibition at different concentrations observed (i.e. 0.7, 0.07 and 0.007 µmol l⁻¹). Clonogenic growth of haematopoietic stem cells was considered to be sensitive if it was ≤ 0.5 × control.

### Statistical analysis

Data were calculated as means and standard deviations of at least three evaluable determinations per data point. Colony survival was calculated by expressing the average number of colony-forming units from cells exposed to each anti-tumour agent relative to the average number of colony-forming units from untreated controls. Results were evaluated statistically by the Friedman repeated measures ANOVA on ranks test. *P*-values ≤ 0.05 were interpreted as indicating significant differences.

The experimental design is summarized in Figure 2.

**Figure 4** Aplidine. Colony growth inhibition. Long-term exposure (21–28 days); median colony growth inhibition at different concentrations

### RESULTS

A total of 56 tumours were included in this experiment. Forty-nine specimens (88%) showed adequate growth in controls using short-term exposure schedules and 54 specimens (96%) showed adequate growth in controls using long-term exposure schedules. The tumour types tested were mainly breast carcinoma, melanoma, ovarian carcinoma, lung carcinoma, colorectal carcinoma, gastric carcinoma and lymphoma.

As shown in Table 1 and Figure 3 for the short-term exposure, aplidine has a profound inhibitory effect on tumour colony formation. The anti-tumour effect is concentration dependent. At 0.01 µmol l⁻¹, in vitro growth of 17/49 (35%) evaluable specimens was inhibited; at 0.05 µmol l⁻¹, 40/49 (82%) specimens were inhibited; and at concentrations ≥ 0.5 µmol l⁻¹, colony formation of 49/49 (100%) tumours was inhibited.

The comparison of median reductions of colony formation also demonstrates that aplidine is active at > 0.01 µmol l⁻¹. Moreover, ovarian, lymphoma and colorectal cancer specimens appear to be selectively sensitive to the lowest concentration, 0.01 µmol l⁻¹, tested in a short-term exposure schedule.

For continuous exposure experiments, concentrations ranged from 0.0001 µmol l⁻¹ to 1.0 µmol l⁻¹. Again, the data show a concentration-dependent anti-tumour activity (Figure 4, Table 2). The lowest active concentration evaluated, 0.001 µmol l⁻¹, suggests that non-small-cell lung cancer, melanoma, and breast cancer might be highly sensitive to this compound at long-term exposure.

The indication that a better activity profile is achievable by using a continuous exposure schedule led to prospective head to head comparisons of short-term vs long-term drug exposures. As shown in Figure 5A–C, the inhibition of tumour-forming units was more pronounced with the long-term exposure. This argues for a schedule-dependent activity of aplidine in vitro.

The toxicity on haematopoietic stem cells is summarized in Tables 3 and 4. The $IC_{50}$ of aplidine appears to be in the range of

© Cancer Research Campaign 1998 

**Table 2** Inhibitory activity of aplidine against tumour colony-forming units from freshly explanted human tumours in vitro using a long-term exposure schedule (21–28 days)

| Tumour type | Aplidine (μmol l⁻¹) | | | | | | |
|---|---|---|---|---|---|---|---|
| | **0.0001** | **0.001** | **0.01** | **0.05** | **0.1** | **0.5** | **1.0** |
| Breast | 0/7[a] | 3/7 | 12/13 | 12/12 | 12/12 | 12/12 | 12/12 |
| Melanoma | 0/10 | 6/10 | 11/12 | 12/12 | 12/12 | 12/12 | 12/12 |
| Ovarian | 0/3 | 0/3 | 11/11 | 11/11 | 11/11 | 11/11 | 11/11 |
| Stomach | 0/1 | 1/1 | 3/3 | 3/3 | 3/3 | 3/3 | 3/3 |
| NSCLC | 1/4 | 3/4 | 4/4 | 3/3 | 3/3 | 3/3 | 3/3 |
| NHL | 1/3 | 1/3 | 4/4 | 3/3 | 3/3 | 3/3 | 3/3 |
| Colorectal | 0/1 | 0/1 | 3/3 | 3/3 | 3/3 | 3/3 | 3/3 |
| Sarcoma | n.d. | n.d. | 2/2 | 2/2 | 2/2 | 2/2 | 2/2 |
| Hodgkin's lymphoma | 0/1 | 1/1 | 1/1 | 1/1 | 1/1 | 1/1 | 1/1 |
| SCLC | n.d. | n.d. | 1/1 | 1/1 | 1/1 | 1/1 | 1/1 |
| Total | 2/30 (7%) | 15/30 (50%) | 52/54 (94%) | 51/51 (100%) | 51/51 (100%) | 51/51 (100%) | 51/51 (100%) |

[a]Number of inhibited specimens (≤ 50% survival of tumour colony forming units) over number of evaluable specimens. n.d., not done.

**Figure 5** In vitro colony growth inhibition with aplidine comparing short- vs. long-term exposure. (**A**) breast cancer. (**B**) melanoma. (**C**) non-small-cell lung cancer

© Cancer Research Campaign 1998

**Table 3** Toxicity of aplidine in comparison with doxorubicin on clonogenic haematopoietic stem cells in vitro using a short-term exposure schedule (1 h)

| Type | Aplidine (μmol l[-1]) | | | Doxorubicin (μmol l[-1]) | | |
|---|---|---|---|---|---|---|
| | 0.01 %S ± s.d.[a] | 0.05 %S ± s.d. | 0.1 %S ± s.d. | 0.007 %S ± s.d. | 0.07 %S ± s.d. | 0.7 %S ± s.d. |
| CFU-GEMM | 97 ± 25 | 40 ± 8 | 49 ± 16 | 65 ± 15 | 76 ± 16 | 0 |
| CFU-GM | 126 ± 27 | 43 ± 10 | 47 ± 7 | 65 ± 8 | 73 ± 5 | 23 ± 2 |
| BFU-E | 90 ± 24 | 52 ± 9 | 48 ± 6 | 99 ± 13 | 99 ± 13 | 8 ± 2 |
| Cluster | 101 ± 25 | 55 ± 11 | 67 ± 11 | 73 ± 11 | 69 ± 21 | 32 ± 4 |
| Inhibitory anti-tumour activity | 35% | 82% | 94% | | | |

[a]Survival (% control) ± standard deviation.

**Table 4** Toxicity of aplidine in comparison with doxorubicin on clonogenic haematopoietic stem cells in vitro using a long-term exposure schedule

| Type | Aplidine (μmol l[-1]) | | | Doxorubicin (μmol l[-1]) | | |
|---|---|---|---|---|---|---|
| | 0.0001 %S ± s.d.[a] | 0.001 %S ± s.d. | 0.01 %S ± s.d. | 0.007 %S ± s.d. | 0.07 %S ± s.d. | 0.7 %S ± s.d. |
| CFU-GEMM | 114 ± 25 | 76 ± 13 | 73 ± 16 | 96 ± 19 | 14 ± 3 | 0 |
| CFU-GM | 99 ± 8 | 98 ± 8 | 40 ± 3 | 107 ± 9 | 4 ± 1 | 0 |
| BFU-E | 98 ± 9 | 89 ± 8 | 45 ± 4 | 91 ± 11 | 3 ± 1 | 0 |
| Cluster | 98 ± 5 | 100 ± 11 | 95 ± 5 | 94 ± 5 | 4 ± 1 | 0 |
| Inhibitory anti-tumour activity | 7% | 50% | 94% | | | |

0.05 μmol l$^{-1}$ for short-term exposure (CFU-GEMM, CFU-GM, BFU-E and cluster). At this concentration, 82% of tumour specimens are inhibited (colony growth ≤ 0.5 × control), with high in vitro activity against breast, melanoma, ovarian, colon, lymphoma and non-small-cell lung cancer. In addition, tumour inhibition at subtoxic concentrations, 0.01 μM, is observed in ovarian and colon cancer and lymphoma. Concentrations in the range of 0.02–0.03 μmol l$^{-1}$ showed similar toxicity to doxorubicin at clinically relevant concentrations. For the long-term exposure, the $IC_{50}$ of aplidine is 0.01 μmol l$^{-1}$ (CFU-GM and BFU-E). At this concentration, between 50% and 94% of the tumours are inhibited (colony growth ≤ 0.5 × control). Moreover, marginally myelotoxic concentrations of aplidine, 0.001 μM, yielded to tumour inhibition in breast, melanoma and non-small-cell lung cancer.

### DISCUSSION

Aplidine is a new marine-derived anti-cancer candidate structurally related to didemnin B, the first marine anti-cancer compound to enter clinical trials (Chun et al, 1986). In fact, didemnin B was the subject of a large phase I/II programme sponsored by the US National Cancer Institute (Bethesda, MD, USA). Initial phase I trials were performed in the early 1980s, but dose escalation was hampered by the onset of severe emesis (Dorr et al, 1988; Stewart et al, 1991). As a result, subsequent phase II studies included suboptimal dose levels, but minor responses were observed in non-small-cell lung cancer and prostatic cancer. Later, a new dose escalation programme with aggressive antiemetic protection was implemented and determined a new maximum tolerable dose and also of new dose-limiting toxicities, specifically cardiac and neuromuscular (Shin et al, 1991). A number of phase II studies incorporating a higher optimal dose were conducted, and positive results in heavily treated patients with low-grade non-Hodgkin's lymphoma (NHL) have recently been presented (Kucuk et al, 1996). The overall data from the phase II programme with didemnin B indicate that repeated cycles of therapy are hampered by acute cardiotoxicity and neurotoxicity. However, the innovative features of the didemnin family as potential anti-cancer entities led to the establishment of a discovery programme searching for active derivatives with differential toxicities and better therapeutic indexes (Faircloth et al, 1995).

Aplidine has been identified as a new generation didemnin that harbours interesting differential patterns in terms of the mechanism of action, potency and toxicological profile (Faircloth et al, 1996). An in vitro study assessing the cardiotoxic potential of aplidine confirms a lack of cardiotoxic effect at active concentrations (Faircloth et al, 1995). In this experiment, the parent compound, didemnin B, shows clear evidence of cardiotoxicity at anti-tumour in vitro concentrations. Moreover, early data from an in vitro study comparing the neurotoxic potential of aplidine and didemnin B, in a murine clonal phaeochromocytoma cell line PC12 (Geldof, 1995), indicate a better therapeutic index for aplidine and confirm the possibility of inducing a marked anti-tumour effect at non-neurotoxic concentrations.

The present report uses an in vitro cloning system with freshly explanted human tumours and clearly shows a concentration-dependent anti-tumour activity. Interestingly, the current work indicates that a better anti-tumour effect is achieved when tumour cells are continuously exposed to the study drug. Moreover, it appears that tumour types such as colon cancer, non-small-cell lung cancer, melanoma, breast and ovarian cancer and lymphomas might be a target for further development. Recent in vivo data confirm activity against human Burkitt's lymphoma and prostatic PC3 androgen-independent tumour models. In addition, the

© Cancer Research Campaign 1998

pattern of tumour inhibition is consistent with a cytostatic effect, supporting a time of exposure–activity relationship in the in vivo setting (Faircloth et al, 1997).

In addition, evaluation of the myelotoxic potential of aplidine has been the subject of the present investigation. Its in vitro haematotoxicity is both affected by concentration and time of exposure. This part of our study suggests the feasibility of improving its therapeutic index by continuous exposure of aplidine, with concentrations of 0.001 μM inducing anti-tumour effects but mild toxicity in bone marrow progenitors. Of note is the observation that myelotoxicity has been a rare event in phase II studies with didemnin B, even at optimal dose levels (Malfetano et al, 1993, 1996; Kucuk et al, 1996).

In summary, the current in vitro investigation identifies a number of tumour types that are sensitive to low concentrations of aplidine. The magnitude of its anti-tumour effect is dependent on the time of exposure and seems to be achievable at non-myelotoxic concentrations. Aplidine is under late preclinical development, and phase I trials incorporating prolonged/protracted infusions are planned.

### ACKNOWLEDGEMENTS

We want to express our gratitude to Eduardo Vega, Pharma Mar Research and Development Biometrics, for his statistical input; Kathleen Meely, Project Leader, Pharma Mar Research and Development, for her critical review of the data; and also to Esther Carrasco, Executive Secretary Pharma Mar Research and Development, for her secretarial and technical assistance during the preparation of this manuscript.

### REFERENCES

Auvinen M (1997) Cell transformation, invasion and angiogenesis: a regulatory role for ornithine decarboxylase and polyamines. *J Natl Cancer Inst* **89**: 533–537

Bradley TR and Metcalf D (1968) The growth of mouse bone marrow cells in vitro. *Aust J Biol Med Sci* **46**: 335

Chun HG, Davies B, Hoth D, Suffness M, Plowman J, Flora K, Grieshaber C and Leyland-Jones B (1986) Didemnin B, the first marine compound entering clinical trials as an antineoplastic agent. *Invest New Drugs* **4**: 279–284

Crews CM, Collins JL, Lane WS, Snapper ML and Schreiber SL (1994) GTP-dependent binding of the antiproliferative agent didemnin to elongation factor 1a. *J Biol Chem* **269**: 15411–15414

Crews CM, Lane WS and Schreiber SL (1996) Didemnin binds to the palmitoyl protein thioesterase responsible for infantile neuronal ceroid lipofuscinosis. *Proc Natl Acad Sci USA* **93**: 4316–4319

Dorr FA, Kuhn JG, Phillips J and Von Hoff DD (1988) Phase I clinical pharmacokinetic investigation of didemnin B, a cyclic depsipeptide. *Eur J Cancer Clin Oncol* **24**: 1699–1706

Faircloth G, Perez J, Fernandez JL, Scheuer P, Avila J, Garcia M, Erba E, D'Incalci M, Cañedo A, Garcia D, Garcia De Quesada T and Jimeno J (1995) Marine depsipeptides with activity against solid tumour models. In *Proceedings 8th ECCO Congress, Paris, 29 October – 2 November*. Abstract no. 122, p. 529

Faircloth G, Rinehart K, Nuñez de Castro I and Jimeno J (1996) Dehydrodidemnin B a new marine derived antitumour agent with activity against experimental tumour models. *Ann Oncol* **7**: 34

Faircloth G, Hanauske A, Depenbroch H, Peter R, Crews C, Manzanares I, Meely K, Grant W and Jimeno J (1997) Preclinical characterization of Aplidine (APD), a new marine anticancer depsipeptide (MADEP). *Proc AACR* **38**(692): 103

Geldof AA (1995) Nerve-growth-factor-dependent neurite outgrowth assay; a research model for chemotherapy-induced neuropathy. *J Cancer Res Clin Oncol* **121**: 657–660

Gomez-Fabre PM, De Pedro E, Medina MA, Nuñez de Castro I and Marquez J (1997) Polyamine contents of human breast cancer cells treated with the cytotoxic agents chlorpheniramine and dehydrodidemnin B. *Cancer Lett* **113**: 141–144

Hanauske A-R, Hanauske U and Von Hoff DD (1985) The human tumour cloning assay in cancer research and therapy. *Curr Probl Cancer* **9**: 1–50

Hanauske U, Hanauske A-R, Marshall MH, Muggia VA and Von Hoff DD (1987) Biphasic effects of vanadium salts on in vitro tumour colony growth. *Int J Cell Cloning* **5**: 170–178

Kucuk O, Young M, Hochster H, Haberman T, Wolf B and Cassileth P (1996) Phase II trial of didemnin B (DDB) in previously treated non-Hodgkin's lymphoma (NHL): an Eastern Cooperative Oncology Group (ECOG) study [ECOG study (Est number 1489)]. *Proc ASCO* **15**: 414

Lobo C, García-Pozo SG, Nuñez de Castro I and Alonso FJ (1997) Effect of dehydrodidemnin B on human colon carcinoma cell lines. *Anticancer Res* **17**: 333–336

Malfetano JH, Blessing JA and Jacobs AJ (1993) A phase II trial of didemnin B (NSC no. 335319) in patients with previously treated epithelial ovarian cancer. *Am J Clin Oncol* **16**: 47–49

Malfetano JH, Blessing JA, Homesley HD, Look KY and McGehee R (1996) A phase II trial of didemnin B (NSC no. 335319) in patients with advanced squamous cell carcinoma of the cervix. *Am J Clin Oncol* **19**: 184–186

Maurer HR and Ali-Osman F (1981) Tumour stem cell cloning in agar-containing capillaries. *Naturwiss* **68**: 381–383

Rinehart KL and Lithgow-Bertelloni A (1991) International Patent Wo/81/04985

Sakai R, Rinehart KL, Kishore V, Kundu B, Faircloth G, Gloer JB, Carney JR, Namikoshi M, Sun F, Hughes RG, García Grávalos D, García de Quesada T, Wilson GR and Heid RM (1996) Structure–activity relationship of the Didemnins. *J Med Chem* **39**: 2819–2834

Shin DM, Holoye PY, Murphy WK, Forman A, Papasozomenos SC, Ki Hong W and Raber M (1991) Phase I/II clinical trial of didemnin B in non-small-cell lung cancer: neuromuscular toxicity is dose limiting. *Cancer Chemother Pharmacol* **29**: 145–149

Stewart JA, Low JB, Roberts J and Blow A (1991) A phase I clinical trial of didemnin B. *Cancer* **68**: 2550–2554

Urdiales JL, Morata P, Núñez De Castro I and Sánchez-Jiménez F (1996) Antiproliferative effect of dehydrodidemnin B (DDB), a depsipeptide isolated from Mediterranean tunicates. *Cancer Lett* **102**: 31–37

Von Hoff DD, Forseth BJ, Huong M, Buchok JB and Lathan B (1986) Improved plating efficiencies for human tumours cloned in capillary tubes versus petri dishes. *Cancer Res* **46**: 4012–4017

© Cancer Research Campaign 1998